# Comparison of Clinical Outcomes of Tracheostomy between COVID-19 and Non-COVID-19 Patients

**DOI:** 10.3390/jcm12237461

**Published:** 2023-12-01

**Authors:** Sung Ha Jung, Joo Hyun Park, HeeJun Yi, Heejung Kim, Gil Joon Lee, Nayeon Choi

**Affiliations:** 1 Department of Otorhinolaryngology-Head and Neck Surgery, Samsung Medical Center, Sungkyunkwan University School of Medicine, Seoul 06351, Republic of Korea; jungsungha@gmail.com (S.H.J.);; 2 Department of Otorhinolaryngology-Head and Neck Surgery, Jeju National University Hospital, Jeju National University College of Medicine, Jeju 63243, Republic of Korea; youryhj@gmail.com; 3 Department of Otorhinolaryngology-Head and Neck Surgery, Myongi Hospital, Hanyang University College of Medicine, Ilsan 10475, Republic of Korea; onlyta28@naver.com; 4 Department of Otorhinolaryngology-Head and Neck Surgery, Kyungpook National University Chilgok Hospital, Kyungpook National University School of Medicine, Daegu 41944, Republic of Korea

**Keywords:** tracheostomy, COVID-19, decannulation, tracheostoma

## Abstract

Background and Objectives: We compared decannulation-related factors between COVID-19 and non-COVID-19 patients who underwent tracheostomy. Subjects and Methods: We conducted a retrospective study of patients who underwent a tracheostomy. The clinical factors were compared between the successful (decannulation within 3 months) and failed decannulation (decannulation over 3 months) groups in COVID-19 and non-COVID-19 patients. Results: The successful decannulation rates were 41.1% in COVID-19 and 45.1% in non-COVID-19 patients, with no significant differences in demographic and clinical factors between the two groups. In the non-COVID-19 patients, the failed decannulation group had a higher proportion of cerebrovascular and pulmonary diseases. Ventilator dependency or increased oxygen demand was the primary cause of decannulation failure in both groups, with no significant differences except for a higher prevalence of swallowing problems in the COVID-19 group (42.4% vs. 20.0%). Conclusions: The predominant cause of decannulation failure was ventilator and oxygen demand in both the non-COVID-19 and COVID-19 patients. In the non-COVID-19 patients, underlying cerebrovascular diseases were considered to have a significant impact on the decannulation process. On the other hand, swallowing problems significantly influenced decannulation among the COVID-19 patients. Therefore, we should consider early and active respiratory and swallowing rehabilitation to facilitate successful decannulation in COVID-19 patients.

## 1. Introduction

The COVID-19 pandemic has created an enormous medical demand, and airway management has imposed an especially heavy workload with a risk of infection to healthcare providers [1,2,3]. COVID-19 has various effects on different organs. It can induce acute respiratory distress syndrome and cardiovascular dysfunction [2,4,5,6]. Almost one-third of critically ill COVID-19 patients revealed decreased systolic function in an echocardiography assessment [4]. Left ventricle systolic dysfunction was associated with septic cardiomyopathy, and right ventricle systolic dysfunction was related to pressure overload due to hypercapnia, positive pressure ventilation, and pulmonary embolism [4]. Additionally, COVID-19 affects the gastrointestinal tract, and some patients experience symptoms such as nausea, vomiting, diarrhea, and abdominal pain. These gastrointestinal symptoms result from direct viral injury, inflammatory response, imbalance in intestinal secretions, and malabsorption [7]. Furthermore, it leads to transient and permanent olfactory dysfunction [8], cutaneous manifestations [9], and coagulation disorders [10].

Among the various organ complications, the most common complication is acute respiratory distress syndrome. Approximately 2.3–22% of patients with COVID-19 require invasive mechanical ventilation through an endotracheal tube [2,3], and the percentage increases to 42% among critically ill patients [5,11]. In addition, 8% to 13% of patients on mechanical ventilation undergo a tracheostomy for ventilation weaning and prolonged intubation [3,5,12,13].

In general, a tracheostomy is recommended within 7 days of mechanical ventilation because it might provide a lower incidence of ventilator-associated pneumonia, mortality, sedation, and laryngotracheal stenosis in survivors and reduce the duration of mechanical ventilation and the hospital stay [14,15,16]. However, the appropriate time for a tracheostomy in COVID-19 patients has been controversial. Some studies have supported an earlier tracheotomy to reduce the intensive care unit stay, while others have reported that a delayed tracheotomy may be more appropriate given the lack of evidence supporting improved outcomes for an earlier tracheotomy [17,18,19].

Most guidelines about tracheostomies in COVID-19 patients have recommended later tracheostomies to minimize the infection risk of health care workers and to allow prognostic information to become clear and the viral load to potentially decrease [20,21,22,23,24]. The American Academy of Otolaryngology–Head and Neck Surgery (AAO-HNS) recommends performing a tracheostomy no earlier than 2 weeks after intubation.

Since the timing of the tracheotomy and the ICU care method are different for COVID-19 patients and non-COVID-19 patients, the factors related to decannulation are considered to be different between COVID-19 and non-COVID-19 patients. As a patient begins to recover from critical complications, medical staff may consider decannulation to facilitate communication and swallowing and prevent tracheostoma-related complications and increased medical burden [25,26]. However, there is a lack of evidence regarding the timing and clinical factors for successful decannulation in COVID-19 patients. Therefore, in this study, we compared the clinical factors between successful and failed decannulation groups among COVID-19 and non-COVID-19 patients and investigated the causes of decannulation failure in a single tertiary hospital.

## 2. Subjects and Methods

### 2.1. Enrolled Patients and Clinical Factors

We retrospectively analyzed patients who underwent tracheostomies between September 2020 and March 2022 at a single tertiary hospital in the Republic of Korea. All the patients included in this study were admitted to the intensive care unit (ICU) for mechanical ventilation. We performed tracheostomies in COVID-19 patients and non-COVID-19 patients during the same study period and conducted a comparative analysis between these two groups. The Institutional Review Board of the authors’ hospital approved this retrospective study (Approval No. 2022-11-030).

All the patients were observed for longer than six months, and we excluded the patients who died within three months after their tracheostomy. All the patients were screened for COVID-19 via polymerase chain reaction of nasopharyngeal swabs.

### 2.2. Definition of Successful and Failed Decannulation

Successful decannulation was defined as decannulation within three months of the tracheostomy. Failed decannulation was defined as inability to remove the tracheostoma within three months of the tracheostomy or a permanent tracheostoma. The enrolled patients were classified into two groups: the successful decannulation group and the failed decannulation group.

The patients expecting decannulation were sent for consultation with the department of otorhinolaryngology—head and neck surgery. We evaluated their vocal fold mobility, saliva pooling, upper airway patency, tracheostoma, suprastoma, and infrastoma using a rigid and flexible nasopharyngeal endoscope. The patients who had aspiration symptoms received a modified barium swallow test for objective evaluation.

The patients who had a patent upper airway without objective and subjective aspiration and no demand for mechanical ventilation and oxygen underwent decannulation. First, the cannula was maintained in a deballooning state for one week, and then a fenestrated cannula was maintained for one week. After that, the cannula was corked. If there were no abnormal findings for one week, the cannula was removed, and the tracheostoma was closed through spontaneous healing without operation.

### 2.3. Clinical Factors Related to Decannulation

Clinical factors were collected, including age, sex, body mass index (BMI, weight (kg)/height (m)^2^), and comorbid disease. The admission period, intubation period, and time between the tracheostomy and successful decannulation were also investigated.

This study was conducted at a tertiary hospital, indicating that a majority of the patients had been transferred from other medical facilities. Consequently, the admission period was calculated from the date of transfer to the present hospital. The intubation procedures were carried out in negative-pressure isolation rooms within the intensive care unit, and the intubation period was defined as the duration from the day of intubation to the day of tracheostomy. To provide a comprehensive understanding of the timeline and outcomes, the time between the tracheostomy and successful decannulation was computed from the day of the tracheostomy to the day of decannulation following examination by an otolaryngologist.

The causes of decannulation failure consisted of ventilator and oxygen dependency, recurrent/prolonged pneumonia, swallowing dysfunction, cardiovascular failure, and impaired cognitive function. Swallowing dysfunction included aspiration symptoms and dysphagia. Patients with subjective complaints of swallowing dysfunction underwent modified barium studies (MBSs), with additional MBSs performed based on clinical progression.

### 2.4. Statistical Analysis

A comprehensive analysis of clinical factors was undertaken, involving a statistical comparison between the non-COVID-19 and COVID-19 groups. Additionally, a detailed exploration extended to comparisons between the groups that experienced successful and failed decannulation. The continuous variables underwent scrutiny via the Mann–Whitney U test, while the categorical variables were meticulously examined using Fisher’s exact test. The threshold for statistical significance was set at a *p*-value less than 0.05. Notably, all the statistical analyses were meticulously executed using SPSS for Windows version 26.0, developed by SPSS Inc. in Chicago, IL, USA.

## 3. Results

### 3.1. Comparison of Clinical Characteristics between Non-COVID-19 and COVID-19 Patients Who Received Tracheostomies

In this study, a total of 82 non-COVID-19 patients were included, while there were 56 COVID-19 patients during the same enroll period (Table 1). The mean age was 66.6 ± 11.7 years in the non-COVID-19 group and 65.5 ± 9.7 years in the COVID-19 group, and it was not significantly different between the two groups (*p* = 0.673). The sex distribution was also not significantly different (*p* = 0.098) between the non-COVID-19 (47 (57.3%) males and 35 (42.7%) females) and the COVID-19 (39 (69.6%) males and 17 (30.4%) females) groups. The body mass index showed no statistical difference (*p* = 0.401) between the non-COVID-19 (25.8 ± 8.0 kg/m^2^) and COVID-19 (24.8 ± 3.6 kg/m^2^) groups. The comorbid diseases consisted of none (*n* = 7, 8.5%), cardiovascular (*n* = 15, 18.3%), cerebrovascular (*n* = 22, 26.8%), pulmonary (*n* = 9, 11.0%), and other disease (*n* = 29, 35.4%) in the non-COVID-19 group and of none (*n* = 8, 14.3%), cardiovascular (*n* = 10, 17.9%), cerebrovascular (*n* = 11, 19.6%), pulmonary (*n* = 6, 10.7%), and other disease (*n* = 21, 37.5%) in the COVID-19 group. The admission period and intubation period were not significantly different (*p* = 0.424 and 0.260, respectively) between the non-COVID-19 (73.2 ± 42.7 and 11.6 ± 13.0 days) and COVID-19 (90.6 ± 47.9 and 9.5 ± 2.9 days) groups. Successful decannulation, which means successful tracheostoma removal within three months, was not significantly different (*p* = 0.106) between the non-COVID-19 (*n* = 37, 60.8%) and COVID-19 (*n* = 23, 41.1%) groups.

### 3.2. Causes of Failed Decannulation

The most prevalent cause of decannulation failure in both the non-COVID-19 group (*n* = 25, 53.3%) and the COVID-19 group (*n* = 19, 57.6%) were ventilator dependency or increased oxygen demand (Table 2). Other causes of failure were recurrent and prolonged pneumonia and poor lung conditions (*n* = 18, 40.0%), impaired cognitive function or cerebrovascular accident (*n* = 13, 28.9%), and cardiovascular failure (*n* = 6, 13.3%) in the non-COVID-19 group. In the COVID-19 group, other failure causes consisted of recurrent and prolonged pneumonia (*n* = 14, 42.4%), impaired cognitive function or cerebrovascular accident (*n* = 11, 33.3%), and cardiovascular failure (*n* = 2, 6.1%). It is important to note that there were no significant differences in the causes of failure between the two groups, except for swallowing problems. Swallowing problems were significantly more prevalent (*p* = 0.031) in the COVID group (*n* = 14, 42.4%) than in the non-COVID group (*n* = 9, 20.0%).

Airway stenosis and vocal fold paralysis were not causes of decannulation failure in either the non-COVID-19 or the COVID-19 patients.

### 3.3. Comparison of Clinical Characteristics between Successful and Failed Decannulation Group in COVID-19 Patients

Successful decannulation was accomplished in 23 (41.1%) patients, while 33 patients (58.9%) had a failed decannulation (Table 3). The mean age for the successful decannulation group was 66.2 ± 9.0 years, and it was 65.9 ± 14.0 years for the failed decannulation group, with no significant difference between the two groups (*p* = 0.341). The distribution of sex also exhibited no significant difference (*p* = 0.992) between the successful group (16 (69.6%) males and 7 (30.4%) females) and the failed decannulation group (23 (69.7%) males and 10 (30.3%) females). The body mass index (BMI) was higher in the failed decannulation groups (28.0 ± 12.6 kg/m^2^) than in the successful decannulation group (23.7 ± 4.1 kg/m^2^), but there was no statistical significance (*p* = 0.641).

Regarding comorbid conditions, the successful decannulation group consisted of patients with various conditions, including none (*n* = 5, 21.7%), cardiovascular (*n* = 3, 13.0%), cerebrovascular (*n* = 5, 21.7%), pulmonary (*n* = 3, 13.0%), and other diseases (*n* = 7, 30.4%). On the other hand, the failed decannulation group included patients with the following comorbidities: none (*n* = 3, 9.1%), cardiovascular (*n* = 7, 21.2%), cerebrovascular (*n* = 6, 18.2%), pulmonary (*n* = 3, 9.1%), and other diseases (*n* = 14, 32.4%). The duration of admission and intubation did not show significant differences (*p* = 0.297 and 0.097, respectively) between the successful decannulation group (74.0 ± 29.4 days and 9.7 ± 2.8 days, respectively) and the failed decannulation group (73.6 ± 64.0 days and 8.5 ± 3.8 days, respectively). In the failed decannulation group, 3 patients (9.1%) achieved decannulation within 3–6 months after tracheostomy, while 30 patients (90.9%) maintained tracheostomy for more than six months.

### 3.4. Comparison of Clinical Characteristics between Successful and Failed Decannulation Groups in Non-COVID-19 Patients

In the non-COVID-19 patients, 37 patients (45.1%) achieved successful decannulation, while 45 patients (54.9%) experienced decannulation failure (Table 4). The mean age for the successful decannulation group was 66.6 ± 11.5 years, and it was 66.2 ± 11.5 years for the failed decannulation group, with no statistically significant difference between the two groups (*p* = 0.797). The distribution of sex also showed no significant variation (*p* = 0.152) between the successful group, consisting of 24 males (64.9%) and 13 females (35.1%), and the failed decannulation group, including 23 males (51.1%) and 22 females (48.9%). The body mass index (BMI) did not exhibit any statistically significant difference (*p* = 0.554) between the successful group (25.4 ± 7.0 kg/m^2^) and the failed group (25.9 ± 8.2 kg/m^2^).

The only statistically significant difference (*p* = 0.047) between the two groups was observed in the composition of comorbid conditions. Comorbid disease in the successful decannulation group was composed of none (*n* = 3, 8.1%), cardiovascular (*n* = 7, 18.9%), cerebrovascular (*n* = 6, 16.2%), pulmonary (*n* = 2, 5.4%), and other diseases (*n* = 19, 51.4%). In contrast, the failed decannulation group, when compared to the successful decannulation group, had a relatively higher proportion of cerebrovascular (*n* = 16, 35.6%) and pulmonary diseases (*n* = 7, 15.6%), and the remaining patients in the failed decannulation group had none (*n* = 4, 8.9%), cardiovascular (*n* = 8, 17.8%), and other diseases (*n* = 10, 22.2%). There were no statistically significant differences (*p* = 0.699 and 0.722, respectively) in the duration of hospital admission and intubation periods between the successful decannulation group (71.2 ± 26.4 days and 11.0 ± 12.0 days, respectively) and the failed decannulation group (77.8 ± 82.1 days and 14.1 ± 12.8 days, respectively). Within the failed decannulation group, there were 17 patients (37.8%) who successfully achieved decannulation between 3 and 6 months, while 28 patients (62.2%) had a prolonged tracheostoma for more than 6 months.

## 4. Discussion

The primary factor contributing to decannulation failure in both the COVID-19 and non-COVID-19 patients was the need for mechanical ventilator support and oxygen in this study. In the non-COVID-19 patients, factors such as underlying conditions, specifically cerebrovascular diseases, were identified as substantial factors in the decannulation process. Conversely, for the COVID-19 patients, it is suggested that post-infection complications, particularly issues related to swallowing such as aspiration and dysphagia, may play a significant role in impeding the decannulation process.

In this manner, the factors influencing decannulation appear to differ between COVID-19 and non-COVID-19 patients, likely attributed to the disparate nature of critical care in these two patient groups. Critically ill COVID-19 patients often face limitations in tests, rehabilitation, and dietary interventions, making it challenging for them to actively engage in nutritional and rehabilitation efforts compared to non-COVID-19 patients. Although not specifically analyzed in our data, it is noteworthy that ICU care for COVID-19 patients frequently involves tube feeding, and oral intake may be deferred until isolation is lifted. Consequently, these factors could contribute to the development of swallowing problems and potentially lead to delayed decannulation. Therefore, when managing patients with respiratory conditions, including those with COVID-19 requiring isolation, it is crucial to be mindful of these aspects for effective clinical care.

The COVID-19 pandemic has led to enormous demands on healthcare systems worldwide, especially in critical care, due to approximately 2–5% of patients with COVID-19 requiring airway management with mechanical ventilation and a tracheostomy [1,2,3,11,12,27].

In our study, demographic and clinical variables including age, sex, BMI, and underlying diseases between the COVD-19 and non-COVID-19 patients were not different. However, decannulation failure due to swallowing problems among the COVID-19 patients was notably high, at 42.4% compared to 20.0% in the non-COVID-19 patients. This is presumed to be associated with delayed oral intake and delayed swallowing evaluation and rehabilitation, as COVID-19 patients tend to minimize medical interventions for prevention of infection among healthcare providers.

Moreover, a higher prevalence of underlying diseases, such as cerebrovascular diseases (35.6%) like brain infarction, was observed in the decannulation failure group among the non-COVID-19 patients. However, in the COVID-19 patients, there was no difference in the prevalence of underlying diseases between the successful and failed decannulation groups. Based on this study, we assumed that post-COVID-19 complications such as aspiration and dysphagia, which affect swallowing, may have significantly impacted decannulation in the COVID-19 patients. Meanwhile, in the non-COVID-19 patients, underlying conditions such as cerebrovascular diseases appeared to play a crucial role in influencing the decannulation process.

In both cohorts of patients, those with COVID-19 and those without, the leading cause of decannulation failure was found to be the dependence on mechanical ventilation or the need for supplemental oxygen, closely followed by recurrent and prolonged pneumonia. Acute respiratory distress syndrome (ARDS) initiates a pathological cascade characterized by diffuse alveolar damage in the lungs [28,29]. In the initial phase, hyaline membrane formation occurs within the alveoli, followed by interstitial widening, edema, and fibroblast proliferation during the subsequent organizing stage [28,29].

The pathological progression of COVID-19-associated ARDS mirrors the typical changes observed in ARDS, notably featuring diffuse alveolar damage in the lungs [30,31]. As patients navigate the course of their illness, recent reports on the extended outcomes of ARDS associated with COVID-19 highlight the emergence of lung fibrosis. A study revealed that approximately 17% of patients exhibited fibrous stripes in chest CT scans, suggesting the potential development of fibrous lesions during the healing phase of pulmonary chronic inflammation or proliferative diseases, which are gradually replaced by scar tissues [31].

Comparative analyses between COVID-19-related ARDS and ARDS from other etiologies highlight the graver outcomes associated with the former. The mortality rates in the intensive care unit and hospital for typical ARDS are reported as 35.3% (95% CI, 33.3–37.2%) and 40.0% (95% CI, 38.1–42.1%), respectively. Conversely, in COVID-19 ARDS, mortality rates range from 26% to 61.5% when admitted to critical care, and among patients receiving mechanical ventilation, mortality can fluctuate between 65.7% to 94%. The identified risk factors for unfavorable outcomes encompass older age, comorbidities such as hypertension, cardiovascular disease, and diabetes mellitus, lower lymphocyte counts, kidney injury, and elevated D-dimer levels. The multifaceted causes of death in COVID-19 ARDS include respiratory failure (53%), respiratory failure combined with cardiac failure (33%), myocardial damage, and circulatory failure (7%), or death from an unknown cause [29]. This comprehensive understanding of the intricate interplay between various factors contributing to decannulation failure and the nuanced progression of ARDS, particularly in the context of COVID-19, underscores the complexity of managing these critically ill patients and informs potential avenues for targeted interventions and improved patient care strategies.

In the era of COVID-19, the indications and timing of tracheostomies in COVID-19 patients have changed compared to those of patients receiving conventional critical care. Most guidelines recommend a later tracheostomy, two weeks after the diagnosis of COVID-19, to lower the infection risk of health care providers and allow time for the patient’s prognosis to become clear [20,21,22]. In this study, we performed tracheostomies relatively early (9.7 ± 2.8 days in the successful decannulation group and 8.5 ± 3.8 days in the failed decannulation group). Since the patients in this study were transferred to critical care after being treated at another hospital, the time to receiving the tracheostomy was relatively short. Likewise, the general clinical situation in the era of COVID-19 has changed, and management of tracheostomies is different from past years. Prolonged intubation in COVID-19 patients might induce voice, swallowing, and airway dysfunction such as vocal cord palsy, dysphagia, and laryngotracheal stenosis, which can impact morbidity and mortality [32,33]. However, there is a lack of knowledge about clinical courses and decannulation in patients with COVID-19. Such analysis is necessary, because tracheostomas increase medical costs and the burden on caregivers. Therefore, in this study, we analyzed the clinical factors between the successful and failed decannulation groups and investigated the causes of failed decannulation. Ventilator dependency and oxygen demand were the most common causes (57.6%) of failed decannulation in the COVID-19 patients in this study. Previous research on decannulation in COVID-19 patients also found that higher oxygen demand and peak cough flow were independent risk factors for delayed decannulation [34].

Recurrent and prolonged pneumonia after COVID-19 infection has been associated with acute respiratory distress syndrome [35], and it was one of the major causes of decannulation failure in this study. However, this was also identified as one of the main causes of decannulation failure in non-COVID-19 patients with a high prevalence.

COVID-19 patients receiving critical care have difficulty with active respiration and swallowing rehabilitation due to limited contact with professional medical staff. Therefore, there is a high possibility of delayed decannulation in COVID-19 patients. The role of swallowing rehabilitation in patients with COVID-19 has not been reported, despite many of these patients presenting with dysphagia in acute care hospitals. Dysphagia is a well-known risk factor for aspiration pneumonia, malnutrition, increased length of hospital admission, and higher mortality [36,37,38,39]. A previous study on dysphagia in COVID-19 patients reported that 28.9% of patients appealed to dysphagia, and swallowing function became normal after active rehabilitation [40]. We also found that many patients suffered dysphagia and aspiration, and 42.4% of the patients failed to achieve decannulation because of aspiration and swallowing problems. Therefore, we expected that active swallowing and respiration rehabilitation could facilitate early decannulation.

When comparing the decannulation success and failure groups in the non-COVID-19 group, a significant difference was observed in the underlying diseases, with cerebrovascular disease accounting for the highest proportion in the failure group at 35.6%. It is believed that patients failing decannulation due to swallowing problems such as aspiration and dysphagia caused by cerebrovascular disease are relatively more prevalent in non-COVID-19 patients, making it a more significant factor in this study. Interestingly, there were no patients with airway stenosis or vocal fold paralysis among those with failed decannulation in our cohort, and these are the major causes of decannulation failure in non-COVID-19 patients.

There are several limitations to our study. This was a retrospective single-center study with a small number of subjects, and it could not establish a definitive conclusion. In addition, most patients were transferred to our hospital after several days of treatment at another hospital. However, we expect that our investigation into decannulation will provide useful information and might allow for appropriate treatment decisions in future outbreaks of respiratory infectious diseases.

In conclusion, ventilator and oxygen demand was the major cause of decannulation failure in both the COVID-19 and non-COVID-19 patients. In the non-COVID-19 patients, underlying conditions such as cerebrovascular diseases are considered to have a significant impact on the decannulation process. On the other hand, it is inferred that post-COVID-19 complications, notably swallowing problems including aspiration and dysphagia, are likely to significantly influence decannulation among COVID-19 patients. Therefore, medical professionals should consider early and active respiratory and swallowing rehabilitation to facilitate successful decannulation in COVID-19 patients.

## Figures and Tables

**Table 1:** Comparison of clinical factors between non-COVID-19 patients and COVID-19 pneumonia patients who received tracheostomies.

	Non-COVID-19	COVID-19	*p*-Value
Patients (*n*, %)	82 (59.4)	56 (40.6)	
Age (years, mean ± SD)	66.6 ± 11.7	65.5 ± 9.7	0.673
Sex (M:F, *n*, %)	47:35 (57.3:42.7)	39:17 (69.6:30.4)	0.098
Body mass index (kg/m^2^, mean ± SD)	25.8 ± 8.0	24.8 ± 3.6	0.401
Underlying disease (*n*, %)			0.777
None	7 (8.5)	8 (14.3)	
Cardiovascular disease	15 (18.3)	10 (17.9)	
Cerebrovascular disease	22 (26.8)	11 (19.6)	
Pulmonary disease	9 (11.0)	6 (10.7)	
Others (DM/HTN/renal/liver disease)	29 (35.4)	21 (37.5)	
Admission period (days, mean ± SD)	73.2 ± 42.7	90.6 ± 47.9	0.424
Intubation period (days, mean ± SD)	11.6 ± 13.0	9.5 ± 2.9	0.260
Decannulation time			
Successful decannulation within 3 months *	37 (45.1)	23 (41.1)	0.106
Failed decannulation (decannulation over 3 months)	45 (54.9)	33 (58.9)	

SD, standard deviation; DM, diabetes mellitus; HTN, hypertension. * Successful decannulation: successful tracheostoma removal within three months.

**Table 2:** Comparison of the causes of decannulation failure between the COVID-19 and non-COVID-19 patients.

Cause of Failure	Non-COVID	COVID	*p*-Value
No. of total patients	82	56	
No. of decannulation failure patients	45 (54.9)	33 (58.9)	
Ventilator dependency or oxygen demand	25 (53.3)	19 (57.6)	0.859
Recurrent and prolonged pneumonia	18 (40.0)	14 (42.4)	0.830
Impaired cognitive function or cerebrovascular accident	13 (28.9)	11 (33.3)	0.104
Swallowing problems	9 (20.0)	14 (42.4)	0.031
Cardiovascular failure	6 (13.3)	2 (6.1)	0.065

“Successful decannulation” was defined as the removal of the tracheostomy tube within 3 months after the tracheostomy procedure, while “decannulation failure” was defined as the presence of a persistent tracheostoma for more than 3 months. Swallowing problems encompassed aspects such as aspiration and dysphagia and were objectively evaluated through modified barium studies.

**Table 3:** Clinical characteristics of patients with COVID-19 who received tracheostomies (*n* = 56).

	Successful Decannulation *	Failed Decannulation ^†^	*p*-Value
Patients (*n*, %)	23 (41.1%)	33 (58.9%)	
Age (years, mean ± SD)	66.2 ± 9.0	65.9 ± 14.0	0.341
Sex (M:F, *n*, %)	16:7 (69.6:30.4)	23:10 (69.7:30.3)	0.992
Body mass index (kg/m^2^, mean ± SD)	23.7 ± 4.1	28.0 ± 12.6	0.641
Underlying disease (*n*, %)			0.587
None	5 (21.7)	3 (9.1)	
Cardiovascular disease	3 (13.0)	7 (21.2)	
Cerebrovascular disease	5 (21.7)	6 (18.2)	
Pulmonary disease	3 (13.0)	3 (9.1)	
Others (DM/HTN/renal/liver disease)	7 (30.4)	14 (32.4)	
Admission period (days, mean ± SD)	74.0 ± 29.4	73.6 ± 64.0	0.297
Intubation period (days, mean ± SD)	9.7 ± 2.8	8.5 ± 3.8	0.098
Decannulation time			
Within 3 months	23 (100)		
3–6 months	-	3 (9.1)	
More than 6 months or permanent tracheostoma	-	30 (90.9)	

SD, standard deviation; DM, diabetes mellitus; HTN, hypertension. * Successful decannulation: successful tracheostoma removal within three months ^†^ Failed decannulation: prolonged tracheostoma for more than three months or permanent tracheostoma.

**Table 4:** Clinical characteristics of non-COVID-19 patients who received tracheostomies (*n* = 82).

	Successful Decannulation *	Failed Decannulation ^†^	*p*-Value
Patients (*n*, %)	37 (45.1)	45 (54.9)	
Age (years, mean ± SD)	66.6 ± 11.5	66.2 ± 11.5	0.797
Sex (M:F, *n*, %)	24:13 (64.9:35.1)	23:22 (51.1:48.9)	0.152
Body mass index (kg/m^2^, mean ± SD)	25.4 ± 7.0	25.9 ± 8.2	0.554
Underlying disease (*n*, %)			0.047
None	3 (8.1)	4 (8.9)	
Cardiovascular disease	7 (18.9)	8 (17.8)	
Cerebrovascular disease	6 (16.2)	16 (35.6)	
Pulmonary disease	2 (5.4)	7 (15.6)	
Others (DM/HTN/renal/liver disease)	19 (51.4)	10 (22.2)	
Admission period (days, mean ± SD)	71.2 ± 26.4	77.8 ± 82.1	0.699
Intubation period (days, mean ± SD)	11.0 ± 12.0	14.1 ± 12.8	0.722
Decannulation time			
Within 3 months	37 (35.1)		
3–6 months	-	17 (37.8)	
More than 6 months or permanent tracheostoma	-	28 (62.2)	

SD, standard deviation; DM, diabetes mellitus; HTN, hypertension. * Successful decannulation: successful tracheostoma removal within three months. ^†^ Failed decannulation: prolonged tracheostoma for more than three months or permanent tracheostoma.

## Data Availability

All data included in this study are available from the corresponding author on reasonable request.
